# Clinical features and enzyme replacement therapy in 10 children with Fabry disease

**DOI:** 10.3389/fped.2023.1084336

**Published:** 2023-02-03

**Authors:** Qian Li, Jing Wang, Minle Tian, Zhenle Yang, Lichun Yu, Suwen Liu, Cong Wang, Xiaoyuan Wang, Shuzhen Sun

**Affiliations:** ^1^ Department of Pediatric Nephrology and Rheumatism and Immunology, Shandong Provincial Hospital, Cheeloo College of Medicine, Shandong University, Jinan, China; ^2^ Department of Pediatric Nephrology and Rheumatism and Immunology, Shandong Provincial Hospital Affiliated to Shandong First Medical University, Jinan, China; ^3^ School of Basic Medical Sciences, Shandong First Medical University, Taian, China

**Keywords:** Fabry disease, children, clinical analysis, screening, enzyme replacement therapy

## Abstract

**Objective:**

To summarize the clinical features, diagnosis and enzyme replacement therapy(ERT) of Fabry disease (FD) in children.

**Methods:**

The clinical data, laboratory tests, genetic variations and treatment of 10 FD children diagnosed in Shandong Provincial Hospital from September 2020 to June 2022 were retrospectively analyzed.

**Results:**

Among the 10 cases from 6 families, 7 patients were boys of 4 to 13 years of age, and 3 were girls of 12 to 15 years of age. There were 7 symptomatic patients, including 6 boys and 1 girl. All 7 patients presented with acral neuralgia. Five patients had little or no sweating. Five patients presented with cutaneous angiokeratoma. Two patients had abdominal pain. One patient developed joint symptoms. Four patients had corneal opacity. One patient had hearing loss; one patient had short stature. One patient had mild proteinuria and 1 patient had dysplasia of the right kidney with decreased eGFR (55.28 ml/min.1.73 m^2^). The left ventricular mass index was slightly elevated in 1 patient. Three patients had mild obstructive ventilatory dysfunction; a small amount of effusion in the intestinal space of the lower abdomen or mild fatty liver was found in 2 patients. Partial empty sella turcica in 1 patient. A total of 6 GLA gene variants were detected in 10 children, among which C.1059_1061delGAT (p.met353del) was a newly discovered mutation. Five children received ERT, of which 4 were treated with agalsidase beta and 1 was treated with agalsidase alpha. Only 1 patient had anaphylaxis. Lyso-GL-3 levels decreased significantly in the first 3 months of ERT initiation and remained relatively stable thereafter in 3 patients. The Lyso-GL-3 level was decreased, but renal impairment continued to progress in 1 patient treated with agalsidase alpha.

**Conclusion:**

The clinical manifestations of FD in childhood are diverse, and it is necessary to make a definite diagnosis by combining family history, enzyme activity, biomarkers, gene testing and other indicators. Pedigree screening and high-risk population screening are helpful for early identification, early diagnosis and early treatment. No serious adverse reactions were found during the short-term treatment with agalsidase alpha and beta.

## Introduction

Fabry disease (FD) is an X-linked inherited lysosomal storage disorder. The GLA gene is located on the long arm of the X chromosome (Q22.1) and encodes alpha-galactosidase A (*α*-Gal A). Mutations in this gene decrease or delete *α*-Gal A activity due to misfolding and modifications. This results in the progressive accumulation of trihexosaccharide sphingolipid alcohol (GL-3) and its derivative deacetyl GL-3 (Lyso-GL-3) in tissues, eventually leading to irreversible damage to multiple organ systems (1). FD was included in China's first list of 121 rare diseases issued in May 2018. The exact prevalence is not known, but the estimated prevalence in the general population is 1 in 100,000. The incidence in neonates is approximately 1/8 882 to 1/1 250, while in male neonates, it is 1/117,000–1/40,000 (2, 3). Due to the insidious onset of the disease in children, diverse clinical manifestations, uncertainty between clinical phenotypes and genotypes, easy misdiagnosis, continuous progression of the disease, and poor prognosis, early diagnosis and treatment in childhood are extremely important. In this study, the clinical characteristics and diagnosis and treatment of 10 children with FD were retrospectively analyzed and summarized to improve clinicians' understanding of FD in children.

## Objects and methods

### Objects

The clinical data of 10 children with FD who were admitted to the Department of Pediatric Nephrology and Rheumatology of Shandong Provincial Hospital from September 2020 to June 2022 were retrospectively analyzed. This study was approved by the Medical Ethics Committee of Shandong Provincial Hospital.

## Methods

### Collection of clinical data

We consulted the medical records of children through the electronic medical record system of the hospital and collected clinical data. The collected information included sex, age, age at diagnosis, time from symptom onset to diagnosis, clinical manifestations, such as acral neuralgia, sweating disorder, cutaneous angiokeratoma, gastrointestinal dysfunction, joint symptoms, eye abnormalities, and hearing abnormalities, routine blood tests, liver and kidney function tests, urine tests, other laboratory tests, electrocardiogram, cardiac color ultrasound, pulmonary function, chest CT, abdominal ultrasound, head magnetic resonance imaging and other auxiliary examinations, enzyme activity levels, Lyso-GL-3 levels, GLA gene variations and treatment options.

### Diagnosis of FD

The clinical manifestations of FD in children lack specificity and are often characterized by acral neuralgia, oligohidrosis, angiokeratoma, gastrointestinal symptoms and corneal opacity. A comprehensive analysis of family history, enzyme activity, biomarkers, pathology and genetic testing is needed to confirm the diagnosis (4).

### Treatment protocol

The current treatment approach for FD includes specific treatment and nonspecific treatment. The former includes enzyme replacement therapy (ERT) and molecular chaperone therapy, and the latter includes symptomatic treatment and psychological support for different organs. There are two kinds of ERT drugs that are available: agalsidase beta (Fabrazyme, 1.0 mg/kg, intravenous infusion every 2 weeks, recommended treatment dose for age ≥8 years) and agalsidase alpha (Replagal, 0.2 mg/kg, intravenous infusion every 2 weeks, recommended treatment dose for age ≥7 years).

### Statistical analysis

Descriptive analysis. Enumeration data are presented as examples, and measurement data are presented as M(range).

## Results

### Clinical manifestations

There were 7 boys and 3 girls from 6 families. The seven boys were diagnosed between the ages of 4 and 13 years. There was one 4-year-old boy who was identified by pedigree screening (case 8). The remaining six boys were between the ages of 11 and 13 years, with a time interval from the first symptom onset to diagnosis ranging from 2 to 9 years. The age range of the three girls was 12–15 years old, and all three of these patients were found by pedigree screening. Seven patients had clinical symptoms, including 6 boys and 1 girl. All 7 patients had acral neuralgia (100%). Five patients (71%) presented with little or no sweating. Five patients (71%) presented with cutaneous angiokeratoma, which was distributed in the fingers, palms, thighs, lower back, buttocks and perineum. Abdominal pain was present in 2 patients (28%). One patient had joint symptoms (14%), involving the bilateral finger joints and knee joints. Corneal opacity was found in 4 patients (57%). Hearing loss was found in 1 patient (14%). One patient had short stature and had been treated with growth hormone (Table 1).

**Table 1:** Clinical manifestations of children with Fabry disease.

Cases	Sex	Age at diagnosis (years)	Interval time (year)	Acral neuralgia	Perspiration disorder	Cutaneous angiokeratoma	Gastrointestinal dysfunction	Arthralgia	Corneal opacity	Hearing loss	Short stature
1	Male	11	7	Yes	No sweat	Yes	No	No	Yes	Yes	No
2	Male	13	8	Yes	Little sweat	No	No	No	No	No	No
3	Male	10	2	Yes	No	Yes	No	No	No	No	No
4	Male	11	2	Yes	Little sweat	No	Abdominal pain	No	Yes	No	No
5	Male	13	9	Yes	Little sweat	Yes	Abdominal pain, diarrhea	Yes	Yes	No	No
6	Male	12	9	Yes	No sweat	Yes	No	No	Yes	ND	No
7	Female	12	2	Yes	No	Yes	No	No	ND	ND	Yes
8	Male	4		No	No	No	No	No	ND	ND	No
9	Female	15		No	No	No	No	No	ND	ND	No
10	Female	15		No	No	No	No	No	ND	ND	No

ND: no data.

### Laboratory examination and auxiliary examination

Of the 7 symptomatic children, 2 were not systematically evaluated in our hospital for personal reasons. Among the other 5 patients, 2 patients had renal involvement. Case 2 showed only mild proteinuria, and the 24-hour urine protein quantification was 0.17 g. After repeated examination, routine urine and microalbumin were normal. Case 4 had dysplasia of the right kidney and right renal artery, mild proteinuria (mainly tubular proteinuria), and decreased eGFR (55.28 ml/min.1.73 m^2^) and was diagnosed as chronic kidney disease stage 3b. One patient had a slightly elevated left ventricular mass index and mild left ventricular hypertrophy (Case 3). Three children had mild obstructive ventilatory dysfunction (Cases 1, 2, and 3). Two cases had mild abdominal ultrasound abnormalities, including a small amount of effusion in the intestinal space of the lower abdomen and mild fatty liver (Cases 1 and 4). Brain magnetic resonance imaging showed partially empty sella turcica in 1 patient (Case 5). The Mainz Severity Score Index (MSSI) was used to score 5 children, with scores ranging from 9 to 21 (Table 2).

**Table 2:** Laboratory examination and MSSI scores of 5 children with Fabry disease.

Case	Blood urea nitrogen (mmol/l)	Serum creatinine (umol/l)	Glomerular filtration rate (ml/min.1.73 m^2^)	Urine protein	Urine microalbumin	Urine red blood cells (cells/HP)	Urine protein creatinine ratio (mg/mg)	24-hour urine protein quantification (g)	Left ventricular mass index (g/m2.7)	Pulmonary function	Abdominal ultrasound	Brain MR	MSSI score
1	3.1	25.1	210.86	Negative	Normal	Normal	0.11	0.09	29.3	Mild obstructive ventilation dysfunction	Small effusion in the lower abdominal intestinal space	Normal	11
2	3.8	36.3	156.86	Negative	Normal	Normal	0.16	0.17	31	Mild obstructive ventilation dysfunction	Normal	Normal	10
3	4.2	31.5	168.02	Negative	Normal	Normal	0.1	0.06	38.4	Mild obstructive ventilation dysfunction	Normal	Normal	9
4	8.2	103	55.28	±∼1+(mixed proteinuria, mainly tubular)	Increased	Normal	0.51	0.31	23.9	Normal	Mild fatty liver, small right kidney and dysplasia of the right renal artery	Normal	21
5	3.9	44.2	125.52	Negative	Normal	Normal	0.07	0.05	22.4	Normal	Normal	Partially empty butterfly saddle	13

### FD-specific indexes and GLA genetic testing

Among the 10 children, serum *α*-gal A enzyme activity was significantly decreased, and serum Lyso-GL-3 levels were significantly increased in 7 boys. Three girls showed normal or mildly decreased enzyme activity with moderately elevated blood Lyso-GL-3 levels. A total of 6 mutations in the GLA gene were found in 10 patients, including 1 nonsense mutation (c.140G > A), 2 deletion mutations (c.1080_1082delTGG and c.1059_1061delGAT), and 3 missense mutations (c.671A > G, c.124A > G, and c.95T > C) (Table 3). Only one mutation was derived from the father, and the rest were derived from the mother. The in-frame deletion variant c.1059_1061delGAT (p.Met353del) is a newly discovered mutation (Case 5) that has not been reported in the literature. This variant is an in-frame deletion mutation that may result in the deletion of the encoded amino acid methionine (Met) at position 353 and was rated as potentially pathogenic according to the ACMG guidelines.

**Table 3:** FD-specific indexes and GLA gene in children with Fabry disease.

Cases	Sex	Blood *α*-Gal A (2.40–17.65 umol/L·h)	Lyso-GL-3 (<1.11 ng/mL)	Exon	GLA gene mutation site	Change in protein sequence	Type of variation	Source of variation
1	Male	0.41	92.98	7	c.140G > A	p.W47X	Nonsense mutation	Mother
2	Male	0.31	96.4	7	c.1080_1082delTGG	p.Gly361del	Deletion mutation	Mother
3	Male	0.31	89.9	7	c.1080_1082delTGG	p.Gly361del	Deletion mutation	Mother
4	Male	0.4	42.58	5	C.671A > G	p.Asn224Ser	Missense mutation	Mother
5	Male	0.29	88.47	7	c.1059_1061delGAT	p.Met353del	In-frame deletion mutations	Mother
6	Male	1.14	69.54	1	c.124A > G	p.Met42Val	Missense mutation	Mother
7	Female	2.03	8.35	1	c.95T > C	p.Leu32Pro	Missense mutation	Father
8	Male	0.32	88.73	7	c.1080_1082delTGG	p.Gly361del	Deletion mutation	Mother
9	Female	2.39	6.18	7	c.1080_1082delTGG	p.Gly361del	Deletion mutation	Mother
10	Female	2.61	1.53	5	C.671A > G	p.Asn224Ser	Missense mutation	Mother

### Pedigree screening

Case 1 was the first FD case admitted to our department. The age at onset of the disease for this patient was 4 years old. In his family, both the mother and the aunt had a history of acroparesthesia at the age of 13–14 years. A maternal GLA gene heterozygous variation was identified, and blood Lyso-GL-3 levels of his mother slightly increased (6.17 ng/ml). His aunt was not screened.

The patients in Case 2 and Case 3 were from the same family. Both of these patients had acroparesthesia. Additionally, one patient had hypohidrosis, and one patient had angiokeratoma. Their maternal grandmothers were siblings, and both had a history of heart disease. The maternal grandmother of Case 3 also had renal dysfunction. Her two brothers died of liver cancer and myocardial infarction at the ages of 42 and 50, respectively. In this family, the mother and sister of Case 2 and the mother and brother of Case 3 were found to carry heterozygous GLA variants, and all were asymptomatic. Serum *α*-Gal A levels decreased slightly and Lyso-GL-3 levels increased slightly in 3 female patients. The younger brother was only 4 years old. His serum *α*-gal A enzyme activity was significantly decreased (0.32 µmol/L·h), and his serum Lyso-GL-3 level was significantly increased (88.73 ng/ml).

The patient in Case 4 presented with intermittent fever with limb pain, little sweating and unexplained abdominal pain at the age of 9. When he was 11 years old, multiple tests were performed. CT examination revealed right kidney atrophy. Additionally, his urine protein was 1+, and serum creatinine was increased (103 µmol/L). All exon parallel genetic testing screening revealed a hemizygous GLA gene mutation; therefore, he was diagnosed with FD. Heterozygous variants of the GLA gene were found in his maternal grandmother, mother, aunt and sister, and they were all asymptomatic, with normal or slightly decreased blood *α*-Gal A levels and slightly increased Lyso-GL-3 levels.

The patient in Case 5 presented with foot pain and arthralgia involving the bilateral finger joints and knee joints since 4 years of age. The patient was misdiagnosed with a cold, growing pains, and juvenile idiopathic arthritis and treated with calcium tablets, nonsteroidal anti-inflammatory drugs, anti-rheumatism medicine, a variety of biological agents, and glucocorticoid treatment without obvious improvement. He was found to have cutaneous angiokeratoma, hypohidrosis symptoms, unexplained abdominal pain and diarrhea since childhood when he came to our department. The patient was diagnosed by screening for FD. His maternal grandmother died of unknown causes in her forties. His mother, maternal uncle and aunt were found to carry the same GLA mutations. His mother had cold finger discomfort when she was approximately 20 years old, and she had a slight decrease in *α*-Gal A levels (1.64 µmol/L·h) and a slight increase in Lyso-GL-3 levels (4.51 ng/ml). His maternal uncle showed acroparesthesia, cutaneous angioglioma, a significant decrease in *α*-Gal A levels (0.24 µmol/L·h), and a significant increase in Lyso-GL-3 levels (58.83 ng/ml). The aunt was asymptomatic, and she had a slight decrease in *α*-Gal A levels (0.58 µmol/L·h) and a slight increase in Lyso-GL-3 levels (8.86 ng/ml).

The patient in Case 6 was diagnosed by screening for FD and presented with hypohidrosis, cutaneous angioglioma, and acroparesthesia at the age of 3 years. Her grandmother had foot pain when she was young, which disappeared after marriage. This family was not screened by experts due to personal factors.

The patient in Case 7 was a girl whose father was diagnosed with FD due to chronic kidney disease, hypertrophic cardiomyopathy, and paroxysmal acroparesthesia. The patient presented with acroparesthesia and was diagnosed with a GLA mutation by screening.

### Treatment

Of the 7 symptomatic patients, two children refused treatment in our hospital for personal reasons and five children were treated with ERT, including 4 with agalsidase beta and 1 with agalsidase alpha. Case 1 was treated with agalsidase beta once every 2 weeks after diagnosis. To date, 35 infusions of agalsidase beta have been used, and no adverse reactions, such as rash, angioedema or blood pressure reduction, have occurred. The patient had a transient exacerbation of acroparesthesia after the fourth infusion of agalsidase beta, which was relieved by oral administration of oxcarbazepine. Up to the follow-up time, the child had been treated with ERT for 1.5 years. During this period, the duration of pain episodes was shorter, and the interval between episodes was longer than it was previously. The child's anhidrosis symptoms were improved, and he reported a sense of dampness in the neck skin. There were no significant changes in cutaneous angiokeratoma or hearing loss. The corneal vortex opacity disappeared after ophthalmic review. The level of Lyso-GL-3 decreased significantly in the first 3 months of ERT initiation and remained between 14.11 and 20.51 ng/ml thereafter.

In Case 2 and Case 3, agalsidase beta therapy was initiated at the time of diagnosis, and in both cases, it had been applied 22 times. Case 2 exhibited anaphylaxis when he was given the third infusion of agalsidase beta. This patient presented with a facial itchy rash, urticaria and hypotension (87/56 mmHg). The symptoms disappeared after adopting dexamethasone and loratadine treatment. Anaphylaxis did not occur when the patient received the following repeated ERT. During the follow-up 12 months after the initiation of ERT, there was no significant relief of acroparesthesia, hypohidrosis, or angiokeratoma in these two patients who were treated with intermittent oral carbamazepine. Case 2 had transient mild proteinuria at the time of diagnosis. However, mild proteinuria (uMALB, U*β*2-ml, U*α*1-M1, uNAG) reoccurred after 12 months, and benazepril and Beling capsules were added for treatment. In Case 3, transient mild proteinuria (urinary protein±, microalbumin 30.27 mg/L) occurred at 6 months after the initiation of ERT. After the addition of benazepril and Beling capsules, the urinary protein and microalbumin levels returned to normal. The levels of Lyso-GL-3 in Cases 2 and 3 also decreased significantly after 3 months of ERT and remained relatively stable thereafter.

Case 4 was treated with agalsidase alpha once every 2 weeks. To date, 7 courses of ERT have been used without adverse drug reactions. This patient had decreased renal function accompanied by proteinuria and was treated with drugs such as ACEI, coenzyme Q10 and polysaccharide iron complex. After 2 courses of treatment, the degree of acroparesthesia was significantly reduced, and abdominal pain did not occur again. However, his serum creatinine level continued to rise to 134 µmol/L, and the serum Lyso-GL-3 level decreased to 11.55 ng/ml.

Case 5 recently started agalsidase beta treatment. To date, 2 courses of ERT have been given, and no adverse reactions have occurred. Antirheumatic drugs and biological agents, oral oxcarbazepine, probiotics and other treatments were stopped. The specific efficacy needs to be further evaluated.

## Discussion

FD can be divided into classic and late-onset types, according to clinical manifestations. Classic FD presents with decreased or absent *α*-Gal A activity and multisystem involvement (brain, kidney, heart, etc.), and the onset of classic FD mostly occurs during childhood (males: 6–10 years old, females: 9–15 years old). Late-onset FD is characterized by a partial decrease in enzymatic activity, and the onset of this type mainly occurs in adulthood. Additionally, the damage is confined to the kidney or heart. Childhood FD is often characterized by acral nerve burning pain, little or no sweat, cutaneous angiokeratoma, proteinuria or microalbuminuria, corneal vorticity, etc (5, 6). Tinnitus, chronic fatigue, and difficulty gaining weight can also occur. Early signs of cardiac involvement (e.g., shortened PR interval, arrhythmias, impaired heart rate variability, valvular sinus aortic dilatation, and mild valvular stenosis) (7) and cerebrovascular abnormalities (involvement of small cerebral vessels) (8) can also occur during adolescence. Although not accompanied by major organ dysfunction, this involvement can lead to physical discomfort and poor school and social performance in children (9). In this study, 7 symptomatic children were diagnosed as classic FD and presented with acral neuralgia. Among these 7 patients, 5 presented with little or no sweat, 5 presented with cutaneous angiokeratoma, 2 presented with abdominal pain, 1 presented with arthralgia, 4 presented with corneal opacity, and 1 presented with hearing loss. Renal involvement occurred in 2 cases. Of these cases, 1 case presented with mild proteinuria, and 1 case presented with right kidney and right renal artery dysplasia, mild proteinuria (mainly tubular proteinuria) and decreased eGFR (55.28 ml/min.1.73 m^2^), which was diagnosed as chronic kidney disease stage 3b. The left ventricular mass index was slightly elevated in 1 case. Three cases had mild obstructive ventilatory dysfunction; a small amount of effusion in the intestinal space of the lower abdomen or mild fatty liver was found in 2 cases by abdominal ultrasound. Magnetic resonance imaging showed partial empty sella turcica in 1 patient. The Mainz Severity Score Index (MSSI) was used to score 5 children with scores ranging from 9 to 21.

Because the clinical manifestations of FD are diverse and nonspecific, most children cannot be diagnosed early after birth or diagnosed immediately after the onset of symptoms. On average, patients with FD see an average of 10 specialists before they are properly diagnosed, and they are often not diagnosed until adulthood. The mean age at diagnosis is 29 years old (10, 11). Reisin R et al. studied 586 FD patients from Europe and other parts of the world and found that the age of diagnosis in children with FD from 2001 to 2006 and from 2007 to 2013 was 7.0 (5.0 to 11.0) years old and 9.0 (6.0 to 11.0) years old, respectively (12). In this study, all 7 symptomatic children had visited other hospitals numerous times. However, due to the atypical clinical symptoms and the lack of understanding of this disease by clinicians, the average age of diagnosis was 11.7 years old (10.0–13.0 years old), and the average delay in diagnosis was 5.57 years (2.0–9.0 years). These findings are close to those of previous reports. Lu ZH et al. reported that two boys with FD were diagnosed at 13.9 years old and 10.9 years old, and the delay of diagnosis was 3 years and 6 years, respectively (13). Zhu XM et al. reported that two boys in two families with FD presented with acroparesthesia at 10 and 14 years old, and the diagnosis was delayed by 6 and 1 years, respectively (14). Therefore, it is very important to improve clinicians' understanding of FD in children for early identification and correct diagnosis of the disease.

Peripheral neuralgia is an early onset, common and suggestive clinical symptom of FD. The main manifestation is acral paroxysmal burning pain, which lasts for several minutes to several weeks. It is often induced by exercise, increased temperature and other factors and is often accompanied by fever and less sweating. Some patients may present with cold or heat hyposensation. FD patients have a marked reduction in myelinated A*δ* fibers (which regulate sharp pain and sense cold), nonmyelinated class C fibers (pain, warm sensation), and nonmyelinated autonomic fibers. The cause of temperature paresthesia is related to small nerve C fibers, which are particularly associated with A*δ* fiber dysfunction (15). Neuralgia in FD can also present as arthralgia. However, due to the heterogeneity of clinical manifestations and insufficient understanding of clinicians, this disease is easily misdiagnosed as growth pains, arthritis, rheumatic fever, dermatomyositis and other diseases. In this study, Case 5 presented with foot pain and arthralgia involving the bilateral finger joints and knee joints since 4 years of age. However, he was misdiagnosed with a cold, growing pains, and juvenile idiopathic arthritis and treated with calcium tablets, nonsteroidal anti-inflammatory drugs, anti-rheumatism medicine, a variety of biological agents, and glucocorticoid treatment without obvious improvement. FD was diagnosed in our center because the patient presented with cutaneous angiokeratoma, acral burning pain, and hypohidrosis, with positive results for enzyme activity, substrates and genes of FD. The deletion variant C.1059_1061delGAT (p.mET353del) in the GLA gene found in this patient's family is a new mutation that has not been reported in the literature. This variant is an in-frame deletion mutation that may result in the deletion of the encoded amino acid methionine (Met) at position 353 and was rated as potentially pathogenic according to the ACMG guidelines. Although the incidence is very low, joint pain needs to be considered a special type of neuralgia in FD by clinicians to avoid misdiagnosis and missed diagnosis to the greatest extent.

As the kidney is the main deposition site and one of the main target organs of GL-3 and the progression to end-stage renal disease (ESRD) is the main cause of death in patients with FD, renal damage has attracted much attention as an important clinical manifestation of FD. GL-3 deposition occurs in almost all cell types of the kidney, including vascular endothelial cells, vascular smooth muscle cells, mesangial cells, mesenchymal cells, podocytes, and distal tubular epithelial cells, and GL-3 deposition begins as early as fetal development (16). Microalbuminuria and increased albuminuria are the first signs of renal impairment, as these conditions occur before the age of 10 years. In typical patients, the glomerular filtration rate (GFR) decreases beginning in puberty (17). With the progression of chronic kidney disease (CKD), albuminuria and massive albuminuria will occur in patients at 20 years of age. Additionally, the severity of renal pathology will increase, and chronic renal impairment will gradually occur and finally develop into ESRD from 30 to 50 years old (18). In this study, 2 cases had renal involvement. Case 2 showed mild proteinuria. Case 4 had dysplasia of the right kidney and right renal artery, mild proteinuria (mainly tubular proteinuria), and decreased eGFR (55.28 ml/min.1.73 m^2^) and was diagnosed with chronic kidney disease stage 3b. The maternal grandmother, mother, aunt and sister of Case 4 harbored the the heterozygous GLA missense mutation C.671A > G (p.Asn224Ser) but were asymptomatic. This locus has been previously reported (19) in a 44-year-old female patient with FD who presented with hypertrophic cardiomyopathy, shortened PR interval, and unexplained syncope. However, her renal function was normal, which was different from the phenotype of this patient. Discordant clinical phenotypes of the same genotype may be associated with the compensatory effects of some related genes, environmental factors, and other factors affecting lysosomal function, which are involved in the occurrence of disease phenotypes (20). Clinicians should carefully consider genetic counseling and prognosis judgment according to family history and genetic testing results, especially in young patients.

FD is an X-linked inherited lysosomal disease; therefore, sex differences have been a concern. The enzyme activity was lower in male patients, mainly in those with the classic type, but the enzyme activity was relatively preserved in female patients, mainly in those with the late type. In this study, we found 13 children and family members with acroneuralgia, including 6 boys, 2 adult males, 1 girl, and 4 adult females. The onset age of pain in boys was between 3 and 9 years old. Most of the female patients were asymptomatic, and a minority of women had acroparesthesia, which began in adolescence and gradually eased with increasing age. Correspondingly, boys and adult males showed significantly reduced enzyme activity and significantly increased substrate levels. Girls and female adults with GLA heterozygous mutations showed normal or slightly decreased enzyme activity and slightly increased substrate levels. Miao YF reported neuralgia in patients with an average starting age of 9 years of age, with an average alleviation at age 20, and a remission rate of 22.8%. Male patients showed more intense pain, and the condition had a greater influence on life in males than in females (21). Male patients with FD tend to progress to ESRD and need dialysis between the ages of 40 to 50 years. However, female heterozygous patients have less renal insufficiency, and the severity is lower than that of male FD. Only a few cases of ESRD in heterozygous women have been reported (22). The severity of disease in female patients varies from asymptomatic to severely involved phenotypes. The occurrence of these conditions is related to the random inactivation of the X chromosome and the severity of pathogenic variants. Echevaria found that an X-chromosome inactivation bias occurred in 29% of 56 female patients with FD, and significant differences in residual enzyme activity, disease severity score, progression in cardiomyopathy, and renal function were associated with different X-chromosome inactivation modes. Therefore, the pattern of X-chromosome inactivation was suggested to significantly affect the phenotype of female patients with FD (23). In addition to these factors, a recent Turkish study showed that coexisting factors might significantly influence the phenotypes of women and men with FD. These factors include elevated levels of lipoprotein (A), homocysteine, total and low-density cholesterol, and antithrombin 3, the prothrombin p.G20210a and factor V Leiden pathogenic variants, and diseases (such as rheumatism or celiac disease) (24). These coexisting factors should be considered in the assessment of patients with FD.

FD usually develops in adolescence and worsens with the progression of the disease. It causes great suffering to patients and their families and imposes a heavy burden on society. As a rare disease, the screening of a high-risk population and family screening can aid in the diagnosis of patients with FD and could result in earlier treatments. If children present with fever accompanied by burning pain of hands and feet, whirlpool cloudy eyes, cutaneous angiokeratoma, and/or the involvement of heart, brain or kidney, and/or a positive family history of disease, they should be screened for enzyme activity and should undergo metabolic substrate screening and genetic screening for FD. Pedigree screening for confirmed patients with FD can help identify potential patients who should receive ERT and other treatments early and improve the prognosis of these patients. According to previous reports, proband-based family screening may detect 3–5 new patients on average. At present, the dry blood disk method (DBS) is often used to screen for FD. Additionally, the activity of *α*-Gal A and Lyso-GL-3 levels in blood can be detected, and GLA gene analysis can be performed simultaneously. In this study, one asymptomatic boy of 4 years of age was identified by pedigree screening, and the remaining 6 cases were identified by the DBS method and/or genetic testing. All 3 girls were identified by pedigree screening. Therefore, it is necessary to improve the understanding of FD and actively carry out high-risk population screening and family screening for the early detection, early diagnosis and treatment of childhood FD.

Enzyme replacement therapy (ERT) has been used in clinical practice since 2001. ERT should be considered for symptomatic boys and girls with neuropathic pain, pathological albuminuria, severe GI involvement and abdominal pain or cardiac involvement. ERT also should be considered for asymptomatic boys with classical (severe) mutations. Timing of ERT depends on individual case for asymptomatic female patients and asymptomatic male patients with late-onset mutations. Organ involvement should be treated as needed (2, 4). Many clinical studies have demonstrated that ERT has positive effects on school attendance, exercise performance, energy level and pain and significantly improves the quality of life of children. The improvement of neuropathic pain with early ERT treatment is more significant and can be maintained in the long term (25). Borgwardt L reported that 10 children between 9 and 16 years of age with FD were treated with agalsidase beta for 1–8 years. During the follow-up period, the symptoms of most patients, such as headache, limb pain and gastrointestinal involvement, were improved (26). However, Mehta A reported (27) that 64 boys and 34 girls with FD were treated with agalsidase alpha for 12 and 24 months. Their renal function and left ventricular mass index remained stable, but there was no significant reduction in the incidence of gastrointestinal symptoms, neuropathic pain, or pain crisis. This study also reported 58 treatment-related adverse events in 23 patients, of which 55 were infusion reactions. In our study, 5 patients were treated with ERT. Of these, 4 patients were treated with agalsidase beta, and 1 was treated with agalsidase alpha. The longest duration of application was 1.5 years, and the shortest duration was 4 weeks. Anaphylaxis occurred in only one case. It presented as a urticaria and hypotension, which quickly subsided after anti-anaphylaxis treatment. Mild proteinuria occurred in case 2 and case 3 at 12 months and 6 months after the initiation of agarsylase *β* respectively. Case 3 received oral benazepril and Beling capsules treatment and proteinuria returned to normal later. Lu ZH et al. reported that one child also experienced transient aggravation of proteinuria after 3 course treated with agarsylase *β* (13). Therefore the changes of proteinuria might be one of the adverse reactions of agarsylase *β*, but the mechanism needs further study. After ERT treatment, acroparesthesia was relieved in 2 children, hypohidrosis was slightly improved in 1 child. Lyso-GL-3 levels decreased significantly in the first 3 months of ERT initiation and remained relatively stable thereafter. This is consistent with previous literature reports (28). However, one child received agalsidase alpha treatment. Although the level of Lyso-GL-3 in blood decreased, the renal function damage continued to progress. Thus, its efficacy needs to be further investigated. Arends M reported a study that enrolled 387 patients. Of these patients, 248 were treated with agalsidase alpha, and 139 were treated with agalsidase beta. After initiation of ERT, plasma Lyso-GL-3 concentrations decreased rapidly and subsequently stabilized in all subgroups. After adjusting for baseline Lyso-GL-3 concentration, sex, and phenotype, the decrease in Lyso-GL-3 levels in patients with classic FD was more pronounced than that in patients treated with *β*-galactosidase (29). However, due to the short duration of ERT treatment in our study, longer follow-up and observation are needed to compare the efficacy and safety of agalsidase *α* and *β*.

In conclusion, FD is a rare X-linked inherited lysosomal storage disease that is prone to misdiagnosis due to its insidious onset and diverse clinical manifestations. For patients with clinical symptoms such as acroparesthesia, little or no sweat, skin angiokeratoma, and corneal opacity, this disease should be highly considered, and the diagnosis should be made based on family history, enzyme activity, biomarkers, gene testing and other indicators. Clinicians should enhance their knowledge and understanding of FD and actively carry out family screening and high-risk population screening, which are helpful for the early identification, early diagnosis and early treatment. No serious adverse reactions were found during the short-term treatment with agalsidase alpha and beta. The comparison of efficacy and safety of agalsidase alpha and beta requires longer periods of follow-up and observation.

## Data Availability

The original contributions presented in the study are included in the article/Supplementary material, further inquiries can be directed to the corresponding author.
